# Associations between left-behind children’s characteristics and psychological symptoms: a cross-sectional study from China

**DOI:** 10.1186/s12888-024-05932-8

**Published:** 2024-07-17

**Authors:** Cong Liu, Yan Xu, Hao Sun, Yan Yuan, Jinkui Lu, Jing Jiang, Ningling Liu

**Affiliations:** 1 Physical Education College, Jiangxi Normal University, Nanchang, China; 2 School of Physical Education, Gannan Normal University, Ganzhou, China; 3 School of Physical Education, Shangrao Normal University, Shangrao, China; 4 Boyue Science Center, Jiangxi Medical College, Shangrao, China

**Keywords:** Left-behind children, Left-behind characteristics, Depression symptoms, Anxiety symptoms, Correlation analysis

## Abstract

**Background:**

Parent-child separation poses a significant challenge for left-behind children (LBC). However, limited empirical evidence exists regarding the correlation between left-behind characteristics and the psychological symptoms of LBC. This study investigated psychological symptoms among LBC and explored associations between left-behind characteristics and those symptoms.

**Methods:**

Using stratified cluster sampling, 1,832 LBC aged 13–18 years from three cities in East China were selected for analysis. Participants’ depression and anxiety symptoms were assessed with the Patient Health Questionnaire 9 and the General Anxiety Disorder 7, respectively. Chi-square tests were used to compare differences in detection rates of psychological symptoms among LBC in different groups. Binary logistic regression analysis was used to infer associations between left-behind characteristics and psychological symptoms.

**Results:**

Depression and anxiety symptoms were detected in 32.86% and 33.24%, respectively, of participating LBC. Univariate analysis showed statistically significant differences in detection rates of depression symptoms by sex, grade, and timing of parent-child separation. Statistically significant differences were observed in anxiety symptom rates by sex, grade, type of caregiver, and timing of mother-child separation. Multivariate analysis indicated a positive association between LBC’s anxiety symptoms and mother-child separation that occurred during post-primary school, and type of caregiver (father only or mother only). Our findings confirm a positive association between left-behind characteristics and anxiety symptoms among LBC.

**Conclusion:**

The timing of mother-child separation and type of caregiver are potential risk factors for the development of anxiety symptoms in this population.

## Background

China’s economy has rapidly developed since it instituted economic reform and the Opening-Up Policy (1978). While people’s living standards have improved, the economic gap between rural and urban areas has expanded since 1990. Many people moved from rural to urban areas to seek employment, but significant numbers of rural children did not accompany or follow their parents to the city; these children are known as left-behind children (LBC) [1]. According to The Ministry of Civil Affairs of China, there were 6.97 million LBC in rural areas at the end of 2018 and this number increased to 11.99 million by the end of 2021 [2].

Being left behind has a complex influence on LBC’s development. On the one hand, better parental employment in urban areas typically increases a family’s income [3]. On the other hand, long-term parental migration adversely affects parent-child relationships, which may negatively impact LBC’s psychological health [4]. A meta-analysis of 89 studies comprising 106,167 LBC aged 0–19 years showed that many of the children had serious psychological health problems, most of which were caused by separating from their parents [5]. Although a few studies have shown nonsignificant differences in the incidence rates of psychological problems among LBC compared with those among non-left-behind children (NLBC) [6], many studies have found a higher risk of psychological problems among LBC than NLBC [7–9]. For example, one study showed that the incidence of serious psychological problems among LBC was approximately 2.7 times higher than that among NLBC, which may be attributable to the interaction effects of multiple factors such as family, school environment, gender, and personality [10]. It is noteworthy that these psychological problems among LBC were diverse, including depression [11], anxiety [9], suicidal ideation [12], neglect [13], and loneliness [14]. In particular, depression and anxiety are the most commonly observed psychological problems among children and adolescents, and are especially prevalent among LBC [15], as indicated by a cross-sectional study, which reported their rates of depression and anxiety disorders as 14.1% and 25.1%, respectively [16]. Another study in Chongqing city investigating 3,107 LBC aged 11–17 years showed that the prevalence of depression and social anxiety among this population group was 19.57% and 12.36%, respectively [17].

It is an indisputable fact that anxiety symptoms (AS) and depression symptoms (DS) have adverse effects on LBC’s health. One study found that major AS and DS are related to some chronic diseases, such as tumors, functional dyspepsia, and chronic viral hepatitis [18]. Other research has corroborated that severe DS in children and adolescents is associated with heightened risks of suicidal ideation, stemming from feelings of hopelessness and intense emotional distress [19]. Therefore, it is crucial to pay attention to LBC’s mental health issues and provide timely interventions.

Several studies have reported that LBC’s psychological health issues are associated with unhealthy lifestyles [20]. One study found, for example, that LBC who habitually skipped breakfast, who had prolonged screen time exposure, and whose sleep patterns were regularly disrupted were more likely to evidence AS [21]. Other studies have indicated they are reportedly also at high risk for psychological problems in school owing to being bullied [22], to discrimination [23], and to sexual assault [24]. Indeed, a longitudinal study has revealed a positive association between being bullied and depression among LBC; this relationship was found to be mediated by negative thoughts and low self-compassion, factors which further increased the risk of depression [22]. Additionally, most research focuses mainly on family factors such as family income [25], parental education [26], and rearing patterns [27]. For instance, some studies have reported that low family income level (family monthly income lower than 2000 RMB) [28] and low parental education level (mother’s education level below high school) [29] were positively associated with severe psychological symptoms (e.g., depression, behavioral problems, and hyperactivity-inattention). Moreover, authoritarian and permissive/neglectful parenting styles reportedly have a significantly negative influence on cognitive-emotional regulation [30].

While extensive studies have analyzed the association between family characteristics and LBC’s psychological symptoms, fewer studies have examined the relationships between LBC’s psychological symptoms and left-behind characteristics (e.g., parent-child separation time, duration of parent-child separation, and type of caregiver). However, strong evidence indicates that left-behind characteristics contain important information about parent-child separation and the living status of LBC, which may effectively predict LBC’s psychological health problems [31, 32]. For example, one study conducted in Jiangxi, China found that timing of parent-child separation after 12 years old exaggerated LBC’s DS [28]. A longer separation has been positively associated with DS among 13–18-year-old LBC [33]. Furthermore, the type of parental migration has been found to influence DS among 10–16-year-old LBC: when both parents migrate, LBC evidence the most depression, compared with LBC of single-parent migrations [34].

Current studies mainly focus on the left behind characteristics of parent-separation time, duration of separation, and type of parental migration. However, the type of caregivers (e.g., mother, father, grandmother, etc.) is also a vital factor that directly influences LBC’s psychological health. The association between the type of caregiver and LBC’s psychological health remains unclear. Moreover, most studies only explore the relationship between left-behind characteristics and DS of LBC; however, general anxiety symptoms are also prevalent among LBC, and must be considered. To address this gap in the existing literature, we used the Patient Health Questionnaire 9 (PHQ-9) and the General Anxiety Disorder 7 (GAD-7) to investigate the detection rates of DS and AS among LBC. In our study, the detection rate represents the percentage of LBC who reported DS and AS. We hypothesized that type of caregivers, parent-child separation status, parent-child separation timing, and the duration of parent-child separation are positively associated with DS and AS among LBC.

## Materials and methods

### Data source and participants

**Fig. 1:**
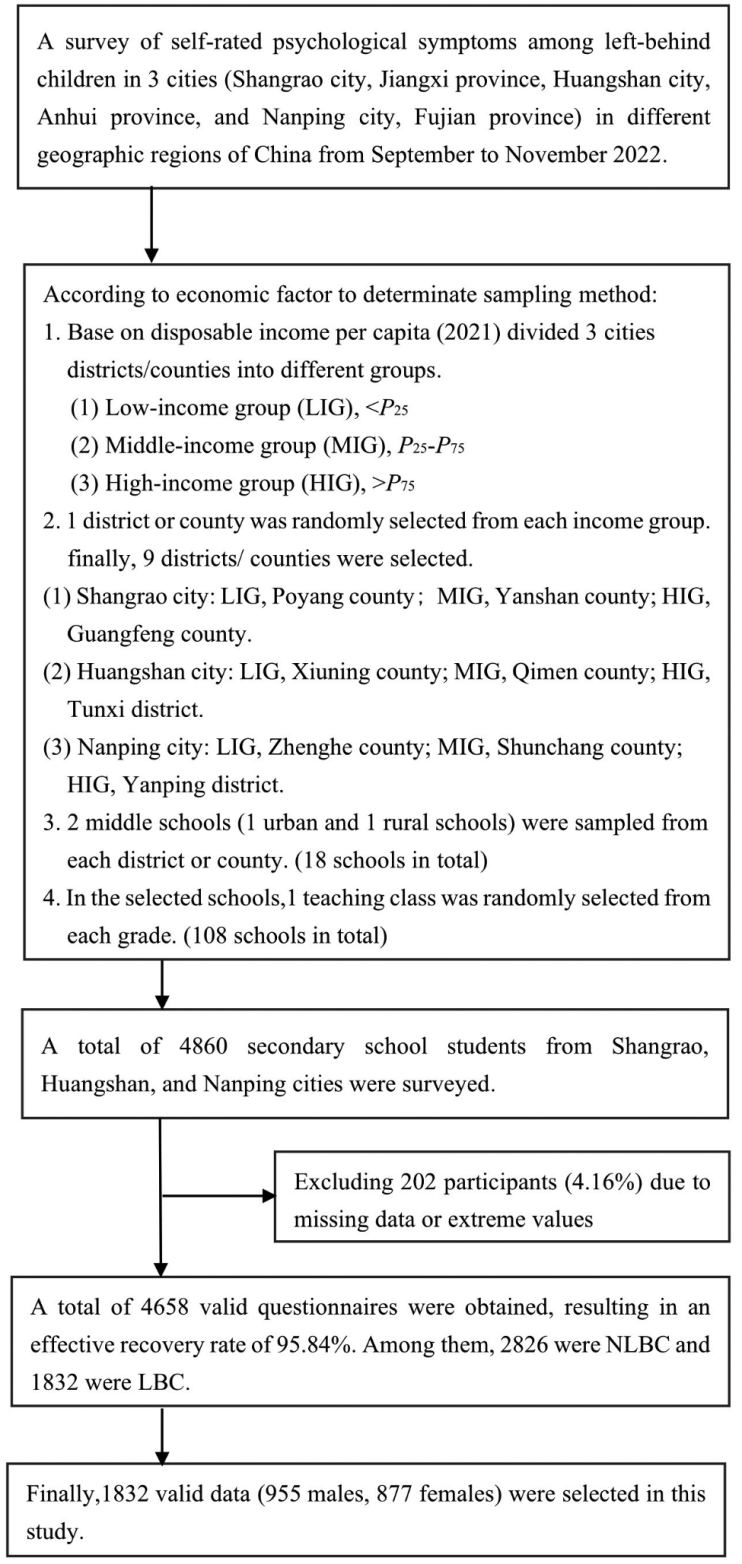
The sampling selection process

Participants were distributed across the Jiangxi, Anhui, and Fujian Provinces in East China. Three cities (Shangrao, Huangshan, and Nanping) were selected from these provinces because of their large migrant populations, as indicated by the 7th National Population Census [35]. The sampling method comprised several steps. First, the districts and counties were divided into a low-income group (LIG), a middle-income group (MIG), and a high-income group (HIG), based on 2021 survey data for each city. Disposable income per capita was used to determine the income groups: LIG < P25, P25 ≤ MIG ≤ P75, and HIG > P75. Second, one district or county was randomly selected from each income group, resulting in a total of nine districts/counties from across the three cities. In Shangrao city, Poyang county was selected for LIG, Yanshan county for MIG, and Guangfeng county for HIG. In Huangshan city, Xiuning county was selected for LIG, Qimen county for MIG, and Tunxi district for HIG. In Nanping city, Zhenghe county was selected for LIG, Shunchang county for MIG, and Yanping district for HIG. Third, In each city, six middle schools were randomly selected, some urban and some rural. One class was then randomly selected from each grade in each of these schools. In total, 18 middle schools, 108 classes, and 4,860 middle school students from the three cities were selected. After excluding 202 participants (4.16%) because of missing data (n = 80, 1.65%) and extreme values (n = 122, 2.51%), we obtained 4,658 valid questionnaires (effective recovery rate 95.84%). Of these, 2,826 were NLBC and 1,832 were LBC. Finally, 1,832 LBC were included as participants in our survey study. The specific criteria for participant enrollment were children and adolescents: (1) aged 13–18 years, with both parents or one parent absent for more than 6 months, (2) who were without congenital organic physical diseases and able to complete the tests for this study, and (3) who voluntarily agreed to participate in this study. The average age of participants was 15.16 ± 1.84 years, 955 (52.1%) were males, 877 (47.9%) were females, 997 (54.4%) attended junior high school, and 835 (45.6%) attended high school. The sampling selection process is shown in Fig. 1.

Before conducting this study, ethical approval was obtained from the medical ethics committee of Jiangxi Medical College [(Rh)2022-1]. Informed consent was obtained from students and their guardians under the ethical approval and their consent to participate. Informed consent forms were signed, and the questionnaire was anonymized to ensure strict privacy protection for study participants.

### Procedures

This study was conducted from September to November 2022. The investigators were trained teachers and students from the School of Physical Education at Shangrao Normal University. All investigators received training and assessment before the survey was administered. The survey was conducted using an online questionnaire, which students completed in their classrooms after classes were finished. The questionnaire took approximately 15 min to complete.

### Questionnaire

The questionnaire collected participants’ basic personal information: sex, grade, and whether they were LBC. It also covered left-behind characteristics: type of caregiver (father or mother, grandparents, or other), parent-child separation status (father-only migration, mother-only migration, dual-parent migration), father-child separation duration (≤ 1 year, 1.1–5 years, ≥ 5.1 years), mother-child separation duration (≤ 1 year, 1.1–5 years, ≥ 5.1 years), parent-child separation duration (≤ 1 year, 1.1–5 years, ≥ 5.1 years), timing of father-child separation (primary or pre-primary school, post-primary school), timing of mother-child separation (primary or pre-primary school, post-primary school), and timing of parents-child separation (primary or pre-primary school, post-primary school). In our study, the left-behind characteristics were categorized on the basis of previous literature [36] and in consideration of the current circumstances.

### DS and AS Screening Tools

#### DS Assessment

The Patient Health Questionnaire 9 (PHQ-9) was used to assess participants’ DS over the previous 2 weeks [37]. The scale contains nine items, each with four response options: “not at all,” “several days,” “more than half the days,” and “nearly every day.” Each item is scored from 0 to 3, with total scores ranging from 0 to 27. A PHQ-9 score of 0–4 indicates no DS, 5–9 indicates mild DS, 10–14 indicates moderate DS, and 15–27 indicates severe DS. In this study, a total PHQ-9 score ≥ 5 was considered an indication of the presence of DS, and scores of 0–4 as no DS. The Cronbach’s α coefficient for the PHQ-9 in a previous study was 0.938 [38]. In our study, the Cronbach’s α coefficient for the PHQ-9 was 0.858.

#### AS Assessment

The General Anxiety Disorder 7 (GAD-7) was used to assess participants’ AS over the previous 2 weeks [39]. The scale comprises seven items, each with four response options: “not at all,” “several days,” “more than half the days,” and “nearly every day.” Each item is scored from 0 to 3, and total scores range from 0 to 21. A score of 0–4 indicates no AS, 5–9 indicates mild AS, 10–14 indicates moderate AS, and 15–21 indicates severe AS. In this study, a total score of ≥ 5 on the GAD-7 was considered an indication of the presence of AS, and scores of 0–4 indicated no AS. The Cronbach’s α coefficient for the GAD-7 in a previous study was 0.928 [40]. In our study, the Cronbach’s α coefficient for the PHQ-9 was 0.896.

### Statistical analysis

The detection rates of DS and AS among LBC with different demographic characteristics were presented using the number and composition ratio. Detection rates were reported using percentages. Chi-square tests were used to compare differences in these rates by group. Binary logistic regression analysis was used to infer associations between left-behind characteristics and DS and AS, and odds ratios (ORs), 95% confidence intervals (CIs), and P-values were obtained, with *P* < 0.05 considered statistically significant. This study hypothesized that different left-behind characteristics were associated with the occurrence of DS and AS in LBC. The data were analyzed and processed using SPSS 22.0 software, and graphs were plotted using GraphPad Prism 9.0.

## Results

In total, 1,832 LBC (males: *n* = 955, 52.13%; females: *n* = 877, 47.87%) participated in our study. The mean age of the surveyed children was 15.16 ± 1.84 years. The detection rate of DS among participants was 32.86% (602/1,832), and that of AS was 33.24% (609/1,832) (see Table 1). There were statistically significant differences in the detection rates of DS by sex, grade, and timing of parent-child separation (χ^2^ = 28.714, 51.159, and 3.889, respectively; *P* < 0.05 for all). Similarly, there were statistically significant differences in the detection rates of AS by sex, grade, type of caregiver, and timing of mother-child separation (χ^2^ = 24.117, 30.464, 8.719, and 5.270, respectively; *P* < 0.05 for all). The analysis by sex showed the detection rates of DS and AS were higher in females (DS: 39.0%; AS: 38.9%) than in males (DS: 27.2%; AS: 28.1%). The analysis by grade showed the detection rates of DS and AS in grades 10–12 (DS: 41.4%; AS: 39.9%) were significantly higher than those in grades 7–9 (DS: 25.7%; AS: 27.7%). Additionally, participants whose caregiver was their father or mother (38.5%) had a higher detection rate of AS than those cared for by grandparents (31.4%) and others (30.7%). Furthermore, in terms of the timing of the parent-child separation or mother-child separation, the detection rates of DS and AS in children who had completed primary school were higher than those in primary or pre-primary school children (DS: post-primary school 42.3%, primary school/pre-primary 33.1%; AS: post-primary school 62.5%, primary school/pre-primary 24.7%).

**Fig. 2:**
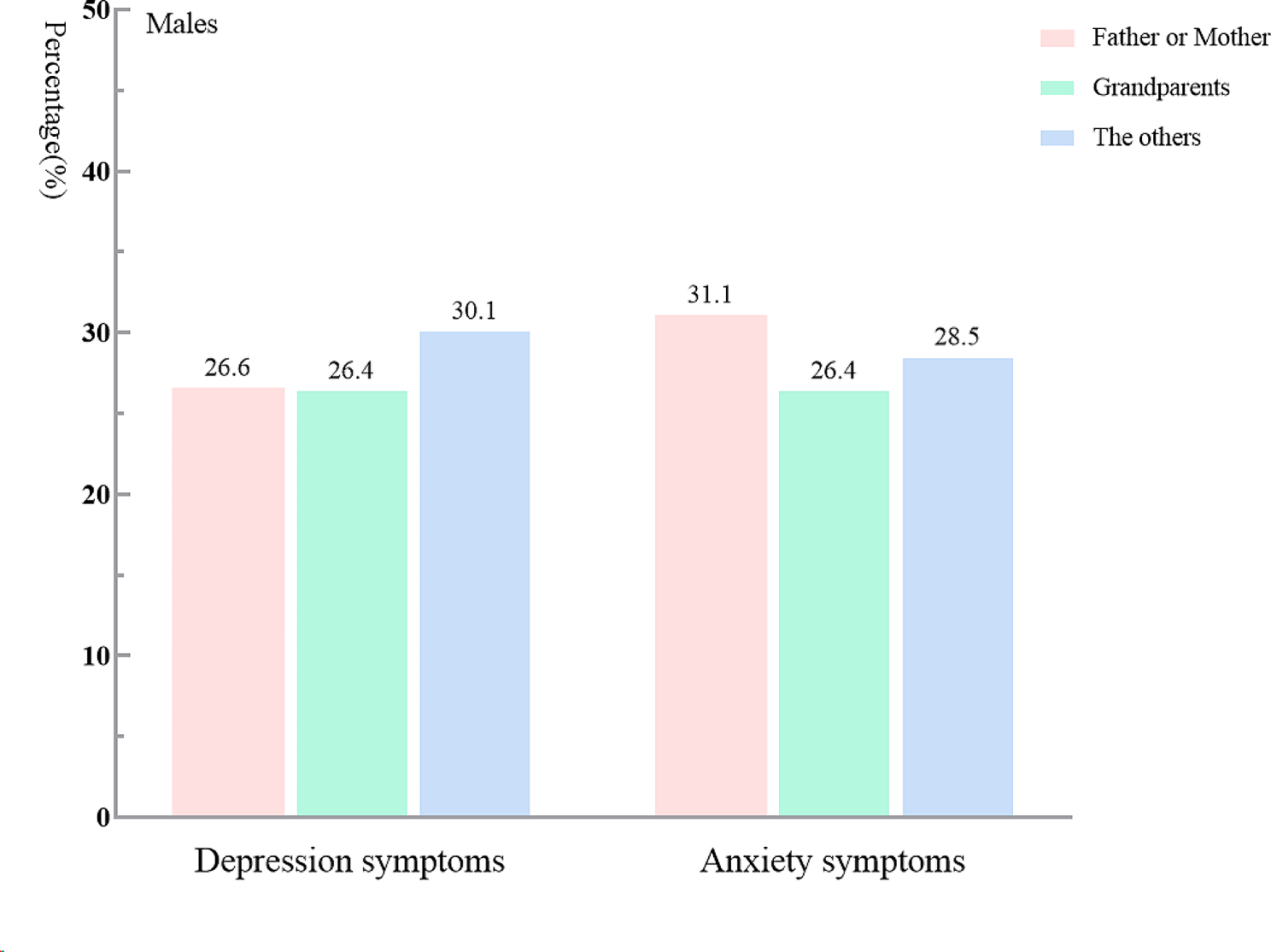
Psychological symptoms of males among LBC according to type of caregiver

**Fig. 3:**
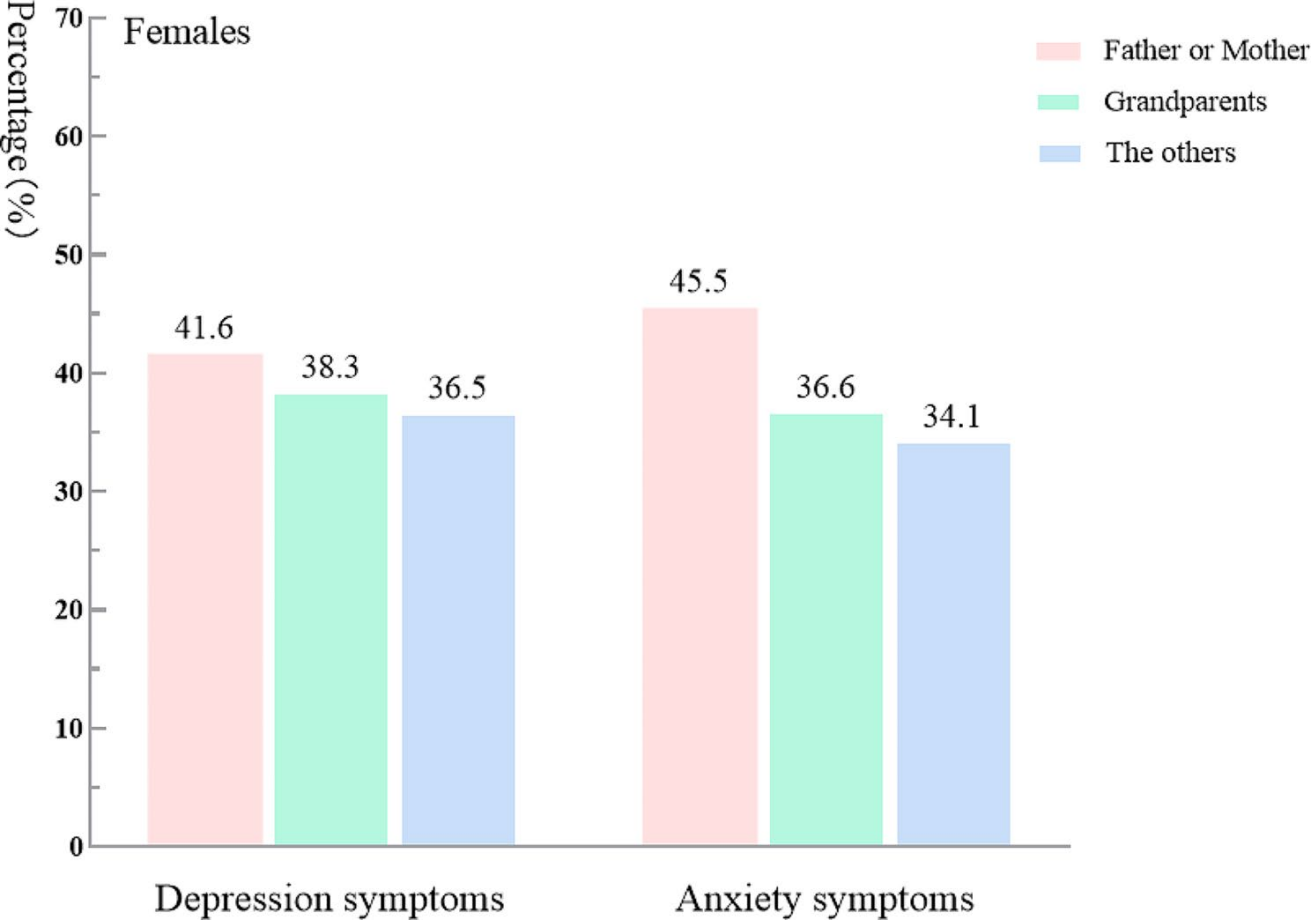
Psychological symptoms of females among LBC according to type of caregiver

**Fig. 4:**
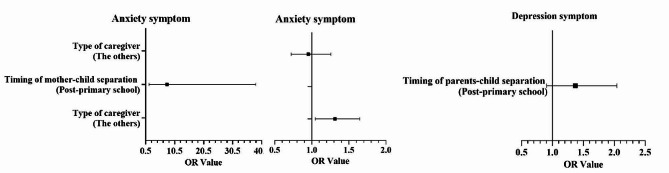
Regression analysis results for the correlation between psychological symptoms and left-behind characteristics

Table 2; Fig. 2 show that, after adjusting for sex and grade, the logistic regression analysis results indicated AS was positively associated with timing of mother-child separation and type of caregiver in post-primary school children (OR = 7.838, 95% CI: 1.599–38.422) and those cared for by their father or mother (OR = 1.312, 95% CI: 1.045–1.647; *P* < 0.05 for all). Figures 3 and 4 show that for both females and males, the AS detection rate among those cared for by their father or mother was more likely to be influenced by the type of caregiver.

**Table 1:** Depression symptoms and anxiety symptoms in left-behind children (*N* = 1,832)

Variables	Total	Depression symptoms			Anxiety symptoms		
		*n*(%)	χ^2^	*P* Value	*n*(%)	χ^2^	*P* Value
**Sex**			**28.714**	**<0.001**		**24.117**	**<0.001**
males	955	260(27.2)			268(28.1)		
females	877	342(39.0)			341(38.9)		
**Grade**			**51.159**	**<0.001**		**30.464**	**<0.001**
Grade 7–9	997	256(25.7)			276(27.7)		
Grade 10–12	835	346(41.4)			333(39.9)		
**Type of caregiver**			0.693	0.707		**8.719**	**<0.05**
Father or Mother	501	172(34.3)			193(38.5)		
Grandparents	1012	326(32.2)			318(31.4)		
The others	319	104(32.6)			98(30.7)		
**Parent-child separation status**			4.676	0.097		1.884	0.390
Father only migration	219	58(26.5)			70(32.0)		
Mother only migration	105	34(32.4)			29(27.6)		
dual-parent migration	1508	510(33.8)			510(33.8)		
**Father-child separation duration**			3.692	0.158		4.724	0.094
≤ 1 year	40	6(15.0)			7(17.5)		
1.1 ~ 5 years	75	20(26.7)			26(34.7)		
5.1 years≥	104	32(30.8)			37(35.6)		
**Mother-child separation duration**			2.592	0.274		5.362	0.068
≤ 1 year	52	13(25.0)			10(19.2)		
1.1 ~ 5 years	31	12(38.7)			9(29.0)		
5.1 years≥	22	9(40.9)			10(45.5)		
**Parents-child separation duration**			2.721	0.256		0.093	0.955
≤ 1 year	882	33(27.0)			41(33.6)		
1.1 ~ 5 years	504	173(34.3)			168(33.3)		
5.1 years≥	122	304(34.5)			301(34.1)		
**Timing of father-child separation**			0.555	0.456		2.065	0.151
Primary or pre-primary school	186	51(27.4)			63(33.9)		
Post-primary school	33	7(21.2)			7(21.2)		
**Timing of mother-child separation**			0.215	0.643		**5.270**	**<0.05**
Primary or pre-primary school	97	32(33.0)			24(24.7)		
Post- primary school	8	2(25.0)			5(62.5)		
**Timing of parents-child separation**			**3.889**	**<0.05**		0.263	0.608
Primary or pre-primary school	1397	463(33.1)			470(33.6)		
Post- primary school	111	47(42.3)			40(36.0)		

**Table 2:** Binary logistic regression analysis results for left-behind characteristics and children’s depression and anxiety symptoms (*N* = 1,832)

Mental symptom	Left-behind characteristics	β-Value	Standard error	Wald χ^2^	OR( 95% CI)	*P*-Value
**Depression symptoms**	**Timing of parents-child separation**					
	Primary or pre-primary school				1	
	Post- primary school	-0.308	0.207	2.206	1.370(0.906 ~ 2.042)	0.137
**Anxiety symptoms**	**Timing of mother-child separation**					
	Primary or pre-primary school				1	
	Post- primary school	2.059	0.811	6.444	7.838(1.599 ~ 38.422)	**<0.05**
	**Type of caregiver**					
	Grandparents				1	
	Father or Mother	0.272	0.116	5.486	1.312(1.045 ~ 1.647)	**<0.05**
	The others	-0.049	0.142	0.118	0.953(0.722 ~ 1.257)	0.731

Note: Adjusted for depression symptoms, anxiety symptoms, sex, and grade

## Discussion

Although many rural-urban migrant workers returned to their hometowns during the COVID-19 pandemic, substantial numbers of LBC still exist in rural areas in China [41]. LBC have suffered severe psychological health issues in recent decades [42, 43]. Given the negative impact that long-term serious psychological health issues have on individuals’ health and well-being [44], it is essential to give adequate attention to the mental health of LBC.

The detection rates of DS and AS among participating LBC in the present study were 32.86% and 33.24%, respectively, which are higher than those for rural NLBC reported in some previous studies [16, 45]. This finding is, however, generally consistent with previous research [46, 47]. One study showed that rates of depression among LBC were higher than those among NLBC, and attributed this to individual lifestyles, that is LBC are more likely to smoke and drink owing to the lack of parental supervision [48]. Another study also associated the prevalence of emotional and behavioral problems among LBC to parental absence; they reported a rate of 28.4%, compared with 21% among NLBC [49]. As these studies illustrate, although LBC’s psychological and health issues are caused by various factors, the main influencing factor is parent-child separation. Previous research has revealed that increasing parent-child communication frequency and duration (more than 5 min each time) will decrease DS among LBC [34]. Therefore, we encourage migration parents to increase their communication frequency and duration with their children, and, importantly, to visit their children regularly.

We examined the relationship between psychological symptoms (DS and AS) and left-behind characteristics in LBC, and found that prolonged mother-child separation that occurred after primary school was positively associated with AS. This finding is consistent with our previous hypothesis in introduction. Our findings highlight the important role that mothers play in their children’s development, especially during adolescence. It has been shown in other work that parental separation during adolescence may increase the risk of AS. Many studies have reported an association between teenagers’ psychological problems and hormones (i.e., their physical development) and learning stress, and suggest that parents play a vital role in teenagers’ psychological counseling [10, 50, 51]. Other studies have shown, moreover, that many teenagers, particularly girls, are likely to seek emotional communication and confide in their mothers [52, 53]. Indeed, mother-child attachment as a special emotional bond has been shown to play a vital role in adjusting children’s negative emotions [54]. However, mother-child separation breaks this attachment for LBC, which may further increase their risk for anxiety [55]. Furthermore, during the middle school period, especially, care received from one’s mother can alleviate low self-esteem, depression, loneliness, and anxiety caused by daily negative events [56, 57]. One study also confirmed that unstable mother-child relationships during middle-school years, compared with those during primary-school years, may predict serious anxiety in teenagers; they attributed this to a lack of social support and communication [58]. Our study further supports these findings by showing that when a mother was absent during adolescence, females have significantly higher levels of AS than males (Figs. 2 and 3). Other research has indicated that females are more susceptible to psychological symptoms, compared with males [59], and parental separation events may aggravate the psychological issues among different LBC (including by sex or gender).

We also found that the type of caregiver (father-child, mother-child, or grandparent-child) was positively associated with AS in LBC, which supports our hypothesis in introduction. Those whose parents were caring for them alone (father-child or mother-child) had a higher risk for AS than those cared for by grandparents (grandparent-child). On the one hand, most studies suggest that LBC raised by their grandparents tend to experience more severe psychological problems than those raised by their parents [60, 61]. This may be because grandparents cannot replace parents in terms of emotional communication. On the other hand, some studies argue that grandparents can form close relationships with LBC, which may help alleviate the children’s psychological problems [62, 63]. There are some possible explanations for this. First, grandparents may provide more comprehensive and attentive care for children than a single parent [64, 65]; most grandparents have a wealth of parenting experience and can provide a stable family environment, which offers an advantage in social support and psychological health among LBC. Second, compared with grandparents, single parents may themselves suffer more severe psychological health issues while raising children owing to financial pressure, lack of support, and separation from a spouse [66]. Studies show that parents’ emotional status relates to their children’s psychological health, and that parents’ adverse emotions increase the risk of depression, anxiety, and suicidal behavior in their children [67–70]. Thus, parental psychological disorders can, arguably, contribute to the development of their children’s social anxiety through a combination of genetic and environmental factors [68]. Single parents may have severe mental health disorders, worsened by rearing children alone, and are then more likely to exhibit neglect, carelessness, and harshness in parenting, which may aggravate the psychological health problems of their LBC. Additionally, when a father or mother is carrying a double burden of work and parenting LBC, they may have less time to pay attention to their children’s psychological symptoms [60, 61].

In this study, we examined the relationship between left-behind characteristics and psychological symptoms among 1,832 LBC from three cities in three provinces in China, which enhanced the objectivity and representativeness of our results. Our findings have practical implications for school-based psychological health education and for promoting healthy development among adolescents. However, this study had some limitations. (1) The measurement of AS was based on a retrospective survey questionnaire, which might have introduced information bias. (2) Parent-child separation duration and parent-child separation timing represent ordinal variables within the context of our analysis. While they offer valuable insights, particularly regarding the temporal dimension of separation experiences, it is notable that compared with continuous variables, they may exhibit a comparatively reduced capacity to capture intricate nuances, potentially leading to a degree of information loss during the analytical phase. (3) The cross-sectional design of this study may limit the accuracy of the results and generalizability of the findings. Further longitudinal studies are needed to accurately assess the strengths of the associations between left-behind characteristics and psychological symptoms among these children.

## Conclusion

This study found that the occurrence of AS among LBC is associated with left-behind characteristics, and the effects of mothers leaving are more significant during adolescence. Furthermore, the risk for developing AS among LBC raised by either their father or mother alone was higher than those raised by grandparents. The timing of mother-child separation and type of caregiver may therefore be a potential risk factor for the development of AS in these adolescents. Schools should pay special attention to psychological health problems that occur during the adolescent stage, as well as to single-parent caregiving situations in this population group.

## Data Availability

To protect the privacy of participants, the questionnaire data will not be disclosed to the public. If necessary, you can contact the corresponding author.

## Abbreviations

AS: Anxiety symptoms
95% CI: 95% confidence interval
DS: Depression symptoms
LBC: Left-behind children
NLBC: Non-left-behind children
OR: Odds ratio
